# Tools for Address Georeferencing – Limitations and Opportunities Every Public Health Professional Should Be Aware Of

**DOI:** 10.1371/journal.pone.0114130

**Published:** 2014-12-03

**Authors:** Ana Isabel Ribeiro, Andreia Olhero, Hugo Teixeira, Alexandre Magalhães, Maria Fátima Pina

**Affiliations:** 1 Instituto de Engenharia Biomédica - INEB, Universidade do Porto, Porto, Portugal; 2 Departamento de Epidemiologia Clínica, Medicina Preditiva e Saúde Pública, Faculdade de Medicina do Porto, Universidade do Porto, Porto, Portugal; 3 Instituto de Saúde Pública da Universidade do Porto - ISPUP, Porto, Portugal; Kenya Medical Research Institute - Wellcome Trust Research Programme, Kenya

## Abstract

Various address georeferencing (AG) tools are currently available. But little is known about the quality of each tool. Using data from the EPIPorto cohort we compared the most commonly used AG tools in terms of positional error (PE) and subjects' misclassification according to census tract socioeconomic status (SES), a widely used variable in epidemiologic studies. Participants of the EPIPorto cohort (n = 2427) were georeferenced using Geographical Information Systems (GIS) and Google Earth (GE). One hundred were randomly selected and georeferenced using three additional tools: 1) cadastral maps (gold-standard); 2) Global Positioning Systems (GPS) and 3) Google Earth, single and in a batch. Mean PE and the proportion of misclassified individuals were compared. Google Earth showed lower PE than GIS, but 10% of the addresses were imprecisely positioned. Thirty-eight, 27, 16 and 14% of the participants were located in the wrong census tract by GIS, GPS, GE (batch) and GE (single), respectively (p<0.001). Misclassification according to SES was less frequent but still non-negligible −14.4, 8.1, 4.2 and 2% (p<0.001). The quality of georeferencing differed substantially between AG tools. GE seems to be the best tool, but only if prudently used. Epidemiologic studies using spatial data should start including information on the quality and accuracy of their georeferencing tools and spatial datasets.

## Background

Health-related events, such as births, diseases and deaths, as well as environmental hazards and socially vulnerable areas, can be located on a map using a terrestrial reference, that is, they can be georeferenced. The exact location of such events help health scientists, in particular epidemiologists, to answer questions involving the word “where?”: “where are people born and where do they live, get sick and die?”, “where are the sources of exposure?”, “where can policy makers intervene to reduce risks or improve access to health services?”

The link between health and geography is not recent. Indeed, one of the first known disease maps dates back to 1789 and was made by Seamon and Pascalis, who georeferenced yellow fever cases in New York [1]. In 1854, John Snow's well-known map of cholera deaths in London became a milestone in modern epidemiology [2].

For centuries maps were almost exclusively produced by cartographers and geographers. The increased use of Geographical Information Systems (GIS) since the late 1980s, plus the larger availability of environmental, socioeconomic and health data, now allows any professional to easily access user-friendly tools to georeference, visualize and analyze spatial data. Address georeferencing (AG) tools have also increased – some are expensive, others freely available, some tremendously complicated and others straightforward. Thus, users need to weigh up the pros and cons of each tool and choose the tool that best suits their research goals. But, at present, there is no complete assessment of the quality of the most widely used AG tools.

The risk of biased findings derived from the inappropriate use of cartographic tools increases proportionally, and directly, with the number of GIS users and spatial epidemiological studies [3], [4]. Errors are particularly frequent during the integration of data from diverse sources, e.g., intersecting address locations with ecological variables. Despite the familiarity of epidemiologists and public health practitioners with concepts such as bias, error and confounding, they have frequently lacked knowledge of the basic concepts of cartography, which (depending on how one deals with them) can “make or break” a GIS investigation [5].

In the present study we aim to compare the different address georeferencing (AG) tools that are currently available with a gold-standard. We evaluate their positional accuracy but, particularly, the frequency of individuals' misclassification using a widely used variable in epidemiologic studies – area-level socioeconomic status. These assessments are conducted using data from a population-based cohort of Porto municipality (Northern Portugal).

### Some basic concepts of cartography and quality of spatial data

**Georeferencing** is usually the first stage in the process of spatial data analysis and it consists of converting a description of a location – for instance an address – to a position on the earth's surface. Georeferencing an address can be made by a pair of coordinates obtained from field survey, either using GPS (Global Positioning System) receivers or topographic instruments, which tend to be more accurate but also time-consuming and expensive; or through computerized systems, using street maps (GIS or online mapping tools such as Google Earth, GE).

Spatial datasets, like any type of data, are prone to errors. Thus, three fundamental concepts have to be kept in mind – precision, bias and accuracy. **Precision** refers to the dispersion of positional random errors and it is usually expressed by a standard deviation. **Bias**, on the other hand, is associated with systematic errors and is usually measured by an average error that ideally should equal zero. **Accuracy** depends on both precision and bias and defines how close features on the map are from their true positions on the ground [6]. So, despite being frequently confused concepts, high precision does not necessarily mean high accuracy. But both depend greatly on the map scale.

All maps have inherent positional errors, which depend on the methods used in the construction of the map. The **scale** is the ratio between a distance on the map and the corresponding distance on the ground. The maximum acceptable positional error (established by cartographic standards) is determined by the map scale. Therefore, the choice of map must take into consideration the scale in which it was created in order to guarantee a positional accuracy that meets the objectives of the study. Some less informed users believe that by zooming in a map they are improving its accuracy and precision. That is not true: accuracy and precision are tied to the original map scale and by zooming in a map within a GIS users are increasing its inherent positional errors.

Address georeferencing also has associated bias, precision and accuracy and its quality depends on the combination of two factors: positional accuracy and completeness [7], [8]. Poor **positional accuracy** might perturb cluster detection and affect the magnitude of regression coefficients – random errors will push coefficients towards the null, whereas systematic ones will underestimate/overestimate associations. **Completeness** is the proportion of records that could be georeferenced and it is evaluated using match rates. Low match rates might reduce statistical power and, eventually, produce biased results due to so-called non-random missingness (match rates differ throughout geographic areas and population strata) [9]. High match rates depend on accurate and detailed address information (known as attribute accuracy and precision) and reference street map.

Some health studies have been conducted using GPS receivers. Be aware, however, that the characteristics of the receivers influence the quality of georeferencing too: the more precise and accurate (positional errors under 1 mm), mostly used in army, engineering and cartography are highly expensive; whereas the most affordable, widely used in epidemiologic studies, have a considerable positional error ranging from 10 to 20 meters.

Knowing the limitations of each spatial dataset is imperative but not enough; usually researchers want to assess the relationships between health data and exposures from the physical and socioeconomic environment, profiting from the potential to combine different spatial data using GIS. GIS inherits the errors from each layer of information. For instance, if the positional accuracy of the AG is 20 meters and we want to overlay a census tract map with a positional accuracy of 5 meters, we could easily fail to pinpoint the participant's address to its actual census tract, as the highest achievable accuracy is that of the least accurate spatial dataset. Eventually, that could lead to so-called **cascading**, as errors propagate from a layer to another, amplifying their effects. Such unpredictable impacts are carefully addressed in the remainder of this article.

## Methods

### Setting

**Figure 1:**
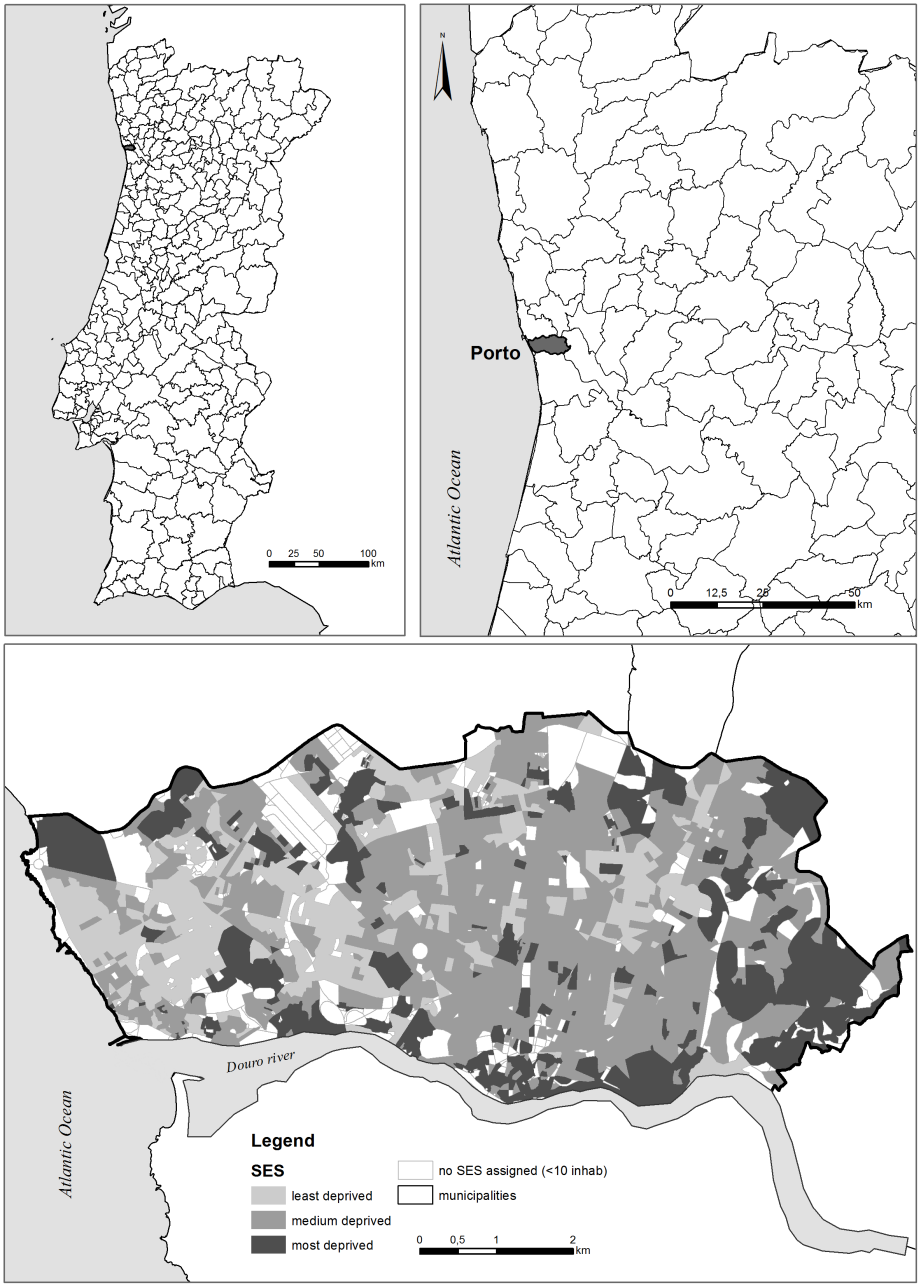
Study Area Location – Porto, Northern Portugal.

Located in the northwest of Continental Portugal, Porto municipality has approximately 250,000 inhabitants distributed across 41.7 km^2^. It is near the Atlantic coast, along the Douro River estuary (Figure 1). Historically, Porto is an industrial and port city and is the second-largest of Portugal. Porto is a homogeneous city in terms of socioeconomic status (SES) - 50% of the population lives in medium SES areas (Figure 1). The spatial distribution of the areas by SES follows a pattern – areas with similar SES tend to be close to each other. Porto also presents a compact urban design (relatively high residential density with mixed land uses).

### Data

We used data from the EPIPorto cohort, which started in 1999 and comprises a random sample of 2485 adults (≥18 years old) living in Porto [10]. Each participant's address of residence was recorded and used for AG. To improve the original address quality, and subsequent georeferencing match rate, all addresses were screened, standardized and parsed.

All subjects gave written informed consent to participate at the time of enrolment. The EPIPorto study protocol was approved by the Local Ethics Committee (São João Hospital) and is in accordance with the Helsinki Declaration principles.

Porto digital map with street centerlines was used as the street reference map for GIS-based AG. Each street segment comprised the following components: direction (‘to’ and ‘from’ node), door number range, name, type (avenue, road, square, etc.) and zip code. Additionally, we acquired a digital map of the census tracts (neighbourhood equivalent) in Porto, then classified according to three discrete classes (from most to least deprived) of socioeconomic status (SES) [11] (Figure 1).

Briefly, that classification was built upon a set of 47 variables available in the 2001 Census at the census tract level. After careful selection (based on statistical criteria and meetings with specialists) the final SES classification included 11 variables relating to the population's age distribution, education level, occupation, and housing conditions (see table below).

To create a summary measure that captured area-level SES, latent class analysis models were run to identify census tracts with similar characteristics. The number of classes was defined according to the Bayesian information criterion, the Akaike information criterion, entropy and interpretability.

Class 1 (least deprived) accounted for 23% of the total number of census tracts. These areas were composed of younger and highly educated populations. Housing conditions were good and housing expenditure was high, whereas unemployment rate was low. Class 2 (medium deprived) accounted for 47% of the census tracts. These areas were composed by older populations with medium education levels. They were characterized by intermediate proportions of damaged buildings, levels of attractiveness and housing expenditure. Finally, class 3 (most deprived) accounted for 30% of the census tracts. These areas were characterized by a medium ageing index and low values of education attainment, employment, attractiveness and housing expenditure.

Census tract's map was used for point-in-polygon overlay operations, in which we attributed a census tract of residence (and corresponding SES) to each participant according to its point positions determined by the different AG tools used.

### Address georeferencing using GIS

All participants, for which addresses were available, were georeferenced using GIS ArcView 9.0 [12] which, by interpolation, places the address in the corresponding street segment and assigns a pair of coordinates. 

Addresses were georeferenced in three phases: 1) automatic, when street map names and the address table names fully matched (spelling score >80%); 2) semi-automatic when the spelling score was <80% and georeferencing was done by interactively selecting from a list of possible locations; 3) manual, when the remaining addresses were georeferenced by searching them in analog maps, placing them in the digital map and retrieving their coordinates. If these approaches failed, participants were contacted to provide correct address information or spatial reference points.

### Google Earth

Addresses were also georeferenced using GE. Three approaches were followed: 1) one address at each time (single GE) in which the user can intervene and pinpoint the address; 2) in a batch (batch GE) using an application which assigns a code to each georeferenced address according to the AG accuracy (exact address, street centroid, building or residential complex centroid or municipality centroid); and 3) in a batch GE without the previous application.

We chose to utilize multiple approaches to consider an important limitation of GE georeferencing: when this tool cannot locate a certain address, it automatically (without alerting the user) searches through other geographical levels (street, municipality, country), until it finds a match, and assigns a pair of coordinates from the centroid of such area. Contrary to what was done for GIS-based AG, addresses that GE could not find and/or precisely georeference were not georeferenced again using manual techniques.

### GPS measurements and field survey

To compare different AG tools in terms of positional error (PE) and misclassification frequency, we selected a random sample of 100 participants from the EpiPorto cohort. Beyond GIS and GE (single and batch), two alternative AG tools were chosen: using GPS receivers and using cadastral maps (ground truth) during field survey. Addresses were distributed evenly by the team and georeferenced using hand-held GPS receivers.

The ground truth location of each address (called here, gold-standard) was assessed by identifying the location on a cadastral map of the city (scale 1/2000). Cadastral maps are detailed maps, which show both natural and built features and are produced with high accuracy standards compatible with large scales (usually between 1/1000 and 1/5000).

Regarding the 100 addresses that were georeferenced by the four AG tools, estimates of the time spent using each tool were quite varied. The assessment of ground-truth and GPS location took 7 days (8 hours each), totaling about 56 hours. Address georeferencing using Google Earth batch tool and Geographic Information Systems took a few minutes, since these are completely automatic tools. Finally, to georeference addresses using Google Earth manual tool, investigators needed about 15 hours.

**Figure 2:**
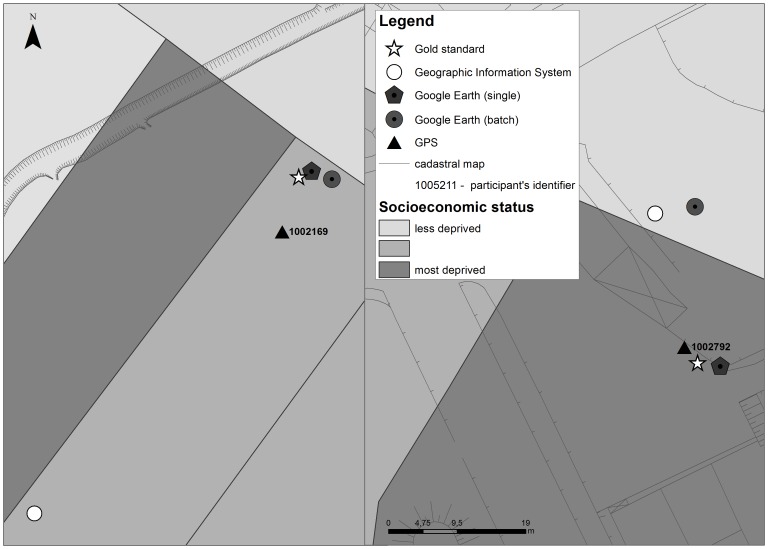
Point position of two participants according to address georeferencing method.

By way of example, Figure 2 depicts the location of two participants according to the AG method.

### Statistical analysis

PE was defined as the Euclidian distance (d), in meters, between the gold standard (x_1_, y_1_) and the locations obtained using the i^th^ other georeferencing tools standard (x_i_, y_i_) (expression 1).
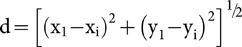
(expression 1)


To characterize PE distributions, descriptive statistics (mean, median, and standard deviation) and boxplots were used. The Friedman test for repeated measures was used to compare median positional error between the different AG tools. Post-hoc analysis with Wilcoxon signed-rank tests was conducted with a Bonferroni correction applied. Cochran's Q test was employed to compare the proportion of misplaced (census tracts) and misclassified (census tract socioeconomic status) individuals between AG tools.

## Results

### Completeness

The EpiPorto baseline database had a total of 2423 addresses, 5 of which were not georeferenced due to incomplete/incorrect addresses, resulting in a match rate of 99.8%. Using GIS, the majority of the records were automatically georeferenced (71.0%) with a smaller proportion by the semi-automatic (13.1%) or manual methods (15.9%).

Using batch GE AG, 84.6% of the addresses were automatically pinpointed in the exact position and 1.9% could not be georeferenced. The remaining addresses were georeferenced at different precision levels (Table 1). Notice that nearly 10% of them were approximately placed (street and municipality centroids).

**Table 1:** Results from Google Earth address georeferencing.

Georeferenced	No. (%)
Exact address	2050 (84.6)
Street centroid	209 (8.6)
Building or residential complex centroid	51 (2.1)
Municipality centroid	66 (2.7)
**Not georeferenced**	**47 (1.9)**
Total	2423 (100.0)

### Positional Error

**Figure 3:**
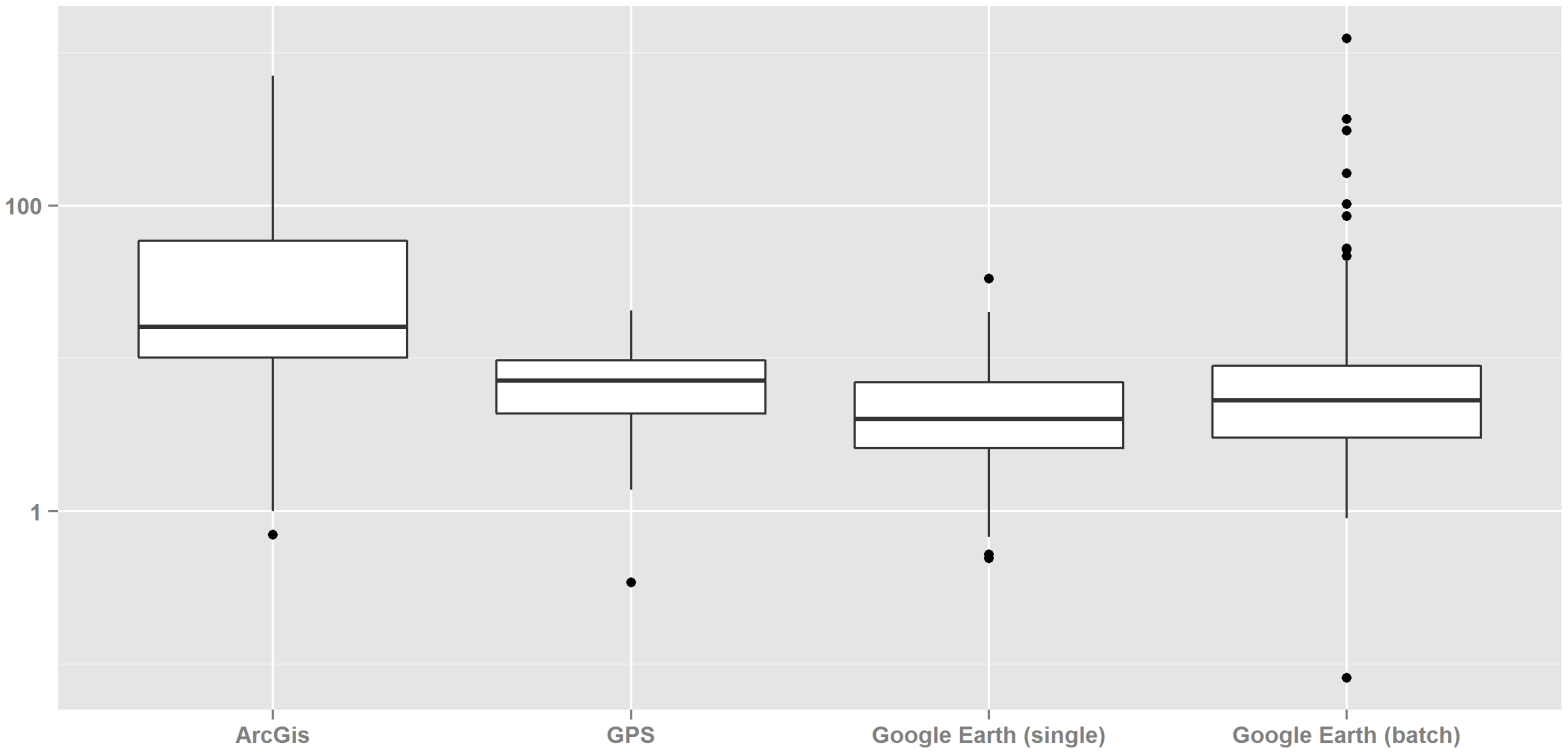
Distribution of positional errors (log-scale) according to address georeferencing method.

We detected statistically significant differences in PE between AG tools (p<0.001) (Table 2 and Figure 3). Compared with all alternative AG tools, median PE using GIS was significantly larger, 16 meters (p<0.001). On the contrary, GE (single) exhibited the best performance, significantly better than GPS (p<0.001). Positional error of methods using a batch of addresses showed highly skewed distributions with maximum positional errors reaching 704 m and 1240 m using GIS and automatic GE, respectively.

**Table 2:** Summary statistics of positional errors (in meters) according to address georeferencing method.

	Median	Mean (SD)	Minimum	Maximum
GIS^a^	16.1	52.0 (100.7)	0.70	704.0
GPS^b^	7.2	7.4 (3.9)	0.34	20.5
Google Earth batch^c^	5.3	30.4 (133.2)	0.1	1240.3
Google Earth single^d^	4.0	5.4 (4.7)	0.0	33.1

a Geographic Information System address georeferencing tool.

b Global Positioning System.

c In a batch using Google Earth address georeferencing tool.

d Address by address using Google Earth address georeferencing tool.

### Misclassification

Thirty-eight, 27, 16 and 14% of the participants were located in the wrong census tract using GIS, GPS, GE (batch) and GE (single) respectively (p<0.001). However, misplaced participants were almost always (more than 96%, regardless the method) positioned in a census tract in the first-order neighborhood, i.e., in a contiguous census tract.

Consequently, because Porto is a homogeneous city, misclassification in census tract SES was less frequent: 14.4, 8.1, 4.2 and 2% using GIS, GPS, GE (batch) and GE (single), respectively (p<0.001). Again, GIS georeferencing showed the worst performance, whereas AG using GE (single) stood out as the best method.

The spatial distribution of misclassified individuals (results not shown) showed no spatial pattern. Misclassification in census tract SES also did not appear differential: the proportion of individuals that were wrongly georeferenced in a less deprived census tract was comparable to the proportion of those that were georeferenced in a more deprived census tract.

## Discussion

In the present study we compared a number of different address georeferencing tools and characterized them according to the following quality criteria: completeness (match rates) and accuracy (positional error and misclassification). Results showed that GE (single) had the highest match rates and the highest accuracy – lower positional error and misclassification – followed by its automatic version (GE batch), GPS and GIS (ArcGis).

Comparing our findings with the literature on this topic was an arduous task: one single European study was found and the remaining were from the USA; they were from large and heterogeneous urban settings; and different AG tools and datasets types were under comparison. Despite such limitations, the positional error we found for GIS AG fell within the previously reported ranges: estimates varied between 200 meters to 10 meters based on mean and median values, always with some extreme outliers [7], [13]–[16]. Notice, however, that most previous studies have used the coordinates obtained using hand-held GPS receivers as the gold-standard – accuracy around 10–20 meters – rather than cadastral maps, which are much more accurate – accuracy of 1 meter for a 1/2000 scale map. Regarding the recently available GE batch AG tool, to our knowledge, only one study addressed its positional error (still relative to GPS measurements) [17]. Authors reported a median error of 22 meters, slightly higher than our estimate. No investigation was undertaken to explore the positional accuracy of single GE (that is, searching addresses one at a time with intervention of the operator) or GPS georeferencing.

Statistics on match rates are much more frequent. Most of the studies reported values around the recommended threshold of 80%; lower match rates are considered unacceptable for epidemiological analysis. However, diverse match rates have been described – from 40 to 99% depending on the type of AG [3], [4], [8], [18]–[23]. AG processes running exclusively in a batch usually lead to low match rates, unless lenient matching options were defined, which would inevitably compromise positional accuracy. In our study, we achieved a match rate (in a batch) of 71% and 85%, respectively for GIS and GE. When GIS AG was used, the 80% requirement was achieved only after semiautomatic and manual approaches. GE match rates, albeit higher, masked important inaccuracies. When we examined the results from the batch GE AG tool, which assigns a code to each address according to the georeferencing accuracy, a reasonable proportion (10%) of the addresses were only approximately placed (street and municipality centroids), leading to extreme outliers of positional error.

Very few studies reported the percentage of misclassified or misplaced addresses. In our analysis we found that a large number of addresses were placed in the wrong census tract, reaching 38% using GIS, which is in accordance with similar studies [4], [24]. Obviously, area misplacements depend on how coarse or fine our territorial units are. Misclassification can be extremely important when using micro-areas, like ours, but inconsequential when using large administrative divisions. Moreover, even when using micro-areas, a non-differential misclassification might not compromise the study findings (although might lead to underestimation of associations), but differential misclassification might lead to biased findings. For instance, in our study, we found no spatial pattern in the position of misclassified individuals and, comparing the SES of the participants' census tracts attributed using GIS and GE (batch) AG, the SES changes were quite random.

Misclassification in census tract (neighborhood) SES was lower, but still non-negligible (14% using the GIS). We found no similar study assessing the misclassification of exposures based on point-in-polygon processes. In our study we observed no differential misclassification, that is, the proportion of individuals that were wrongly georeferenced in a less deprived census tract was comparable to the proportion of those that were georeferenced in a more deprived census tract. However, investigations attempting to determine to what extent misclassification of contaminant exposure affects epidemiological analysis found that the misclassification is extremely high for this kind of small area analysis [7], [13].

Some limitations of our study must be highlighted. Firstly, our findings are based on a single urban setting. Porto is a relatively homogeneous city in terms of the physical and socioeconomic environment. This means that results could be generalizable to other medium-sized urban settings, but not to larger cities or rural areas. However, our study fills a gap in the scientific literature of studies undertaken in medium sized urban settings, especially in Europe, where space is more fragmented and geographical units are, consequently, much smaller. Also hampering generalization, our reference data (street centerlines and census tracts) have their own positional accuracy, which will undoubtedly differ from the ones employed in other contexts. The same extends to the georeferencing tools we used. Secondly, we only examined the misclassification for a single environmental determinant – neighbourhood SES, composed of three levels with a patterned spatial distribution across the municipality. Nevertheless, neighborhood SES is considered in almost every multilevel epidemiological study and the distribution of neighborhood SES tends to be spatially patterned in most urban settings (deprived areas stand near each other like the affluent areas). Our findings are therefore useful for the critical evaluation of results from these studies.

## Conclusions

In the present study we aimed to inform epidemiologists and public health practitioners about the fundamental concepts of cartography and demonstrate the advantages and drawbacks of some currently available address georeferencing techniques. Address georeferencing tools differed significantly and the recently available Google Earth batch tool was revealed to be a valuable alternative method relative to GIS, but only if prudently used. There were a considerable amount of misclassified and misplaced addresses, which were universal to all address georeferencing tools. Our results also suggest misclassification errors were random, i.e., non-differential. However, future studies should assess the effect of AG inaccuracies in determining exposures to other area-level determinants (e.g. air pollution, noise, ambient temperature), especially in Europe where spatial analysis has become frequent, but has not been accompanied by methodological assessments on spatial data quality. Further studies are also needed to evaluate the impact of participant's misclassification (regarding a wide range of variables from the physical and socioeconomic environment) on subsequent statistical analysis and conclusions.

## References

[1] (1834) Rapport sur la marche et les effets du choléra dans Paris et le département de la Seine: année 1832. Paris: Impr. royale

[2] John S (1855) On the mode of communication of cholera London: John Churchill.

[3] Oliver MN, Matthews KA, Siadaty M, Hauck FR, Pickle LW (2005) Geographic bias related to geocoding in epidemiologic studies. Int J Health Geogr 4:29. 16281976 10.1186/1476-072X-4-29 PMC1298322

[4] Griffith DA, Millones M, Vincent M, Johnson DL, Hunt A (2007) Impacts of Positional Error on Spatial Regression Analysis: A Case Study of Address Locations in Syracuse, New York.

[5] Foote K, Huebner D (2000) Error, Accuracy, and Precision. The Geographer's Craft Project, Dept of Geography, University of Colorado at Boulder.

[6] Aronoff S (1989) Geographic information systems: a management perspective. USA: Wdl Pubns.

[7] Zandbergen PA (2007) Influence of geocoding quality on environmental exposure assessment of children living near high traffic roads. BMC Public Health 7:37. 17367533 10.1186/1471-2458-7-37 PMC1838415

[8] Zandbergen PA (2008) A comparison of address point, parcel and street geocoding techniques. Computers, Environment and Urban Systems 32:214–232.

[9] Vach W (1997) Some issues in estimating the effect of prognostic factors from incomplete covariate data. Stat Med 16:57–72. 9004383 10.1002/(sici)1097-0258(19970115)16:1<57::aid-sim471>3.0.co;2-s

[10] Santos AC, Barros H (2003) Prevalence and determinants of obesity in an urban sample of Portuguese adults. Public Health 117:430–437. 14522159 10.1016/S0033-3506(03)00139-2

[11] Alves L, Silva S, Severo M, Costa D, Pina MF, et al (2013) Association between neighborhood deprivation and fruits and vegetables consumption and leisure-time physical activity: a cross-sectional multilevel analysis. BMC Public Health 13:1103. 24289151 10.1186/1471-2458-13-1103 PMC3879067

[12] ESRI (2004) ArcView 9.0 edition. Redlands, California.

[13] Zandbergen PA, Green JW (2007) Error and bias in determining exposure potential of children at school locations using proximity-based GIS techniques. Environ Health Perspect 115:1363–1370. 17805429 10.1289/ehp.9668 PMC1964899

[14] Schootman M, Sterling DA, Struthers J, Yan Y, Laboube T, et al (2007) Positional accuracy and geographic bias of four methods of geocoding in epidemiologic research. Ann Epidemiol 17:464–470. 17448683 10.1016/j.annepidem.2006.10.015

[15] Vieira VM, Howard GJ, Gallagher LG, Fletcher T (2010) Geocoding rural addresses in a community contaminated by PFOA: a comparison of methods. Environ Health 9:18. 20406495 10.1186/1476-069X-9-18 PMC2867955

[16] Zhan FB, Brender JD, De Lima I, Suarez L, Langlois PH (2006) Match rate and positional accuracy of two geocoding methods for epidemiologic research. Ann Epidemiol 16:842–849. 17027286 10.1016/j.annepidem.2006.08.001

[17] Quesada JA, Nolasco A, Moncho J (2013) Comparación de las aplicaciones de Google y Yahoo para la geocodificación de direcciones postales con fines epidemiológicos. Revista Española de Salud Pública 87:201–206. 23775108 10.4321/S1135-57272013000200009

[18] Whitsel EA, Quibrera PM, Smith RL, Catellier DJ, Liao D, et al (2006) Accuracy of commercial geocoding: assessment and implications. Epidemiol Perspect Innov 3:8. 16857050 10.1186/1742-5573-3-8 PMC1557664

[19] Goldberg DW, Wilson JP, Knoblock CA, Ritz B, Cockburn MG (2008) An effective and efficient approach for manually improving geocoded data. Int J Health Geogr 7:60. 19032791 10.1186/1476-072X-7-60 PMC2612650

[20] McElroy JA, Remington PL, Trentham-Dietz A, Robert SA, Newcomb PA (2003) Geocoding addresses from a large population-based study: lessons learned. Epidemiology 14:399–407. 12843762 10.1097/01.EDE.0000073160.79633.c1

[21] Howe HL (1986) Geocoding NY State Cancer Registry. Am J Public Health 76:1459–1460. 10.2105/ajph.76.12.1459-b PMC1646958 3777300

[22] Wey CL, Griesse J, Kightlinger L, Wimberly MC (2009) Geographic variability in geocoding success for West Nile virus cases in South Dakota. Health Place 15:1108–1114. 19577505 10.1016/j.healthplace.2009.06.001 PMC2752286

[23] Zandbergen PA, Chakraborty J (2006) Improving environmental exposure analysis using cumulative distribution functions and individual geocoding. Int J Health Geogr 5:23. 16725049 10.1186/1476-072X-5-23 PMC1523259

[24] Krieger N, Waterman P, Lemieux K, Zierler S, Hogan JW (2001) On the wrong side of the tracts? Evaluating the accuracy of geocoding in public health research. Am J Public Health 91:1114–1116. 11441740 10.2105/ajph.91.7.1114 PMC1446703

